# Preliminary Evaluation of Protective Efficacy of Inactivated Senecavirus A on Pigs

**DOI:** 10.3390/life11020157

**Published:** 2021-02-18

**Authors:** Yuwan Li, Yangyi Zhang, Yingxin Liao, Yawei Sun, Yang Ruan, Chenchen Liu, Mengru Zhang, Fangfang Li, Xiaowen Li, Shuangqi Fan, Lin Yi, Hongxing Ding, Mingqiu Zhao, Jindai Fan, Jinding Chen

**Affiliations:** 1 College of Veterinary Medicine, South China Agricultural University, Guangzhou 510642, China; waner20191028012@stu.scau.edu.cn (Y.L.); zhangyy6@mail.sustech.edu.cn (Y.Z.); yxliao@soil.gd.cn (Y.L.); syw18530494979@stu.scau.edu.cn (Y.S.); ruanyang@stu.scau.edu.cn (Y.R.); liuchenchen@stu.scau.edu.cn (C.L.); zmr15625156296@stu.scau.edu.cn (M.Z.); fangfangli@stu.scau.edu.cn (F.L.); xiaowenlee@stu.scau.edu.cn (X.L.); shqfan@scau.edu.cn (S.F.); yilin@scau.edu.cn (L.Y.); dinghx@scau.edu.cn (H.D.); zmingqiu@scau.edu.cn (M.Z.); 2 Guangdong Laboratory for Lingnan Modern Agriculture, College of Veterinary Medicine, South China Agricultural University, Guangzhou 510642, China; 3 Key Laboratory of Zoonosis Prevention and Control of Guangdong Province, Guangzhou 510642, China

**Keywords:** Senecavirus A, phylogenetic analysis, pathogenicity, protective efficacy, inactivated vaccine

## Abstract

Senecavirus A (SVA), formerly known as Seneca Valley virus (SVV), causes vesicular symptoms in adult pigs and acute death of neonatal piglets. This pathogen has emerged in major swine producing countries around the world and caused significant economic losses to the pig industry. Thus, it is necessary to develop strategies to prevent and control SVA infection. Herein, an SVA strain (named GD-ZYY02-2018) was isolated from a pig herd with vesicular symptoms in Guangdong province of China in 2018. The present study aimed to carry out the phylogenetic analysis of the GD-ZYY02-2018 strain, determine its pathogenicity in finishing pigs, and assess the protective efficacy of the inactivated GD-ZYY02-2018 strain against virus challenge. The results of phylogenetic analysis showed that the SVA GD-ZYY02-2018 strain belonged to the USA-like strains and had a close genetic relationship with recent Chinese SVA strains. Animal challenge experiment showed that 100-day-old pigs inoculated intranasally with SVA GD-ZYY02-2018 strain developed vesicular lesion, low fever, viremia, and virus shedding in feces. The immunization challenge experiment showed that pigs vaccinated with inactivated GD-ZYY02-2018 strain could produce a high titer of anti-SVA neutralizing antibody and no vesicular lesion, fever, viremia, and virus shedding in feces was observed in vaccinated pigs after challenge with GD-ZYY02-2018 strain, indicating that inactivated GD-ZYY02-2018 could protect finishing pigs against the challenge of homologous virus. In conclusion, preliminary results indicated that inactivated GD-ZYY02-2018 could be used as a candidate vaccine for in-depth research and might be conducive to the prevention and control of SVA infection.

## 1. Introduction

Senecavirus A (SVA), also known as Seneca Valley virus (SVV), belongs to the genus *Senecavirus* in the family *Picornaviridae*. SVA is a non-enveloped, single-strand, positive-sense RNA virus and its genome contains a 5’-untranslated region (UTR), a large open reading frame (ORF), and a 3’-UTR. The ORF encodes a polypeptide, which is cleaved into twelve mature proteins including the leader protein (L), four structural proteins (VP1, VP2, VP3, and VP4), and seven non-structural proteins (2A, 2B, 2C, 3A, 3B, 3C, and 3D) [1]. SVV-001, the SVA prototype strain, was originally isolated from cell culture media and identified as the cell culture contaminant in the USA in 2002. Studies about SVV-001 focused on its oncolytic activity in cancer therapy [1]. In recent years, SVA infection of pigs, which was associated with porcine vesicular disease, has been reported in the USA, Canada, Brazil, China, and other regions of the world [2,3,4]. The clinical signs caused by SVA were clinically indistinguishable from those caused by foot and mouth disease virus (FMDV), swine vesicular disease virus (SVDV), vesicular stomatitis virus (VSV), and vesicular exanthema of swine virus (VESV) [5]. These clinical signs mainly included fluid-filled/ruptured vesicles and ulcerative lesions at the snout, coronary band, and hooves, as well as anorexia and lameness in adult pigs [6]. Meanwhile, SVA infection could result in increased mortality in neonatal piglets [2,7]. 

In 2015, the first Chinese SVA strain was isolated from pig herd with vesicular symptoms in Guangdong province [8]. Characteristic clinical symptoms including vesicular lesions in infected sows and acute death of infected piglets were observed. After that, more and more cases of SVA infection were reported in other provinces of China, which meant that SVA was rapidly and widely spread in China [9,10]. The wide spread and presence of SVA strains resulted in the emergence of novel variants. It was reported that there were different types of SVA strains circulating in China [11]. Furthermore, variation and recombination of SVA strains might lead to changes in viral immunogenicity and virulence. Currently, there are no well-established strategies to limit the spread of SVA. Therefore, continuous epidemiology investigation and pathogenicity studies on novel strains are important for surveillance of the state of SVA in pig herds. For effectively prevention and control of SVA infection, vaccine development for SVA is also worth considering.

Continuous monitoring of the prevalence of SVA in Guangdong province of China is of great significance, because Guangdong province is a major pig-raising province and the first Chinese SVA strain was isolated from Guangdong province. In the present study, an SVA strain from Guangdong province was isolated in 2018. This study aimed to carry out the phylogenetic analysis of the newly identified SVA strain, determine its pathogenicity, and assess the protective efficacy of inactivated SVA strain against virus challenge. This study will contribute to the prevention and control of SVA infection.

## 2. Materials and Methods

### 2.1. Cells and Clinical Samples

Baby hamster kidney 21 (BHK-21) cells were grown in Dulbecco’s modified Eagle’s medium (DMEM) complemented with 10% (v/v) fetal bovine serum (FBS) at 37 °C in a humidified 5% CO_2_ incubator.

In May 2018, an outbreak of the vesicular disease occurred in a pig farm in Guangdong province of China. Vesicular fluid and coronary band tissue samples were collected from the clinically affected pigs and stored at −80 °C until they were used for RNA extraction and virus isolation.

### 2.2. RNA Extraction and RNA Detection for Pathogens

The viral RNA in the samples was extracted using a Viral RNA Kit (OMEGA Bio-Tek, Norcross, GA, USA). The extracted RNA was reverse transcribed into cDNA using M-MLV reverse transcriptase (Takara, Dalian, China) according to the manufacturer’s instruction. A pair of primers (SVA-F: 5′-CCTCAGAGACACAGAACTC-3′ and SVA-R: 5′-GAAAGGGTGATCGGGAAG-3′) designed by Primer Premier 6.0 was used to detect SVA. The PCR amplification was performed in a 20 μL volume containing 2× Taq Mix (10 μL), ddH_2_O (7 μL), the forward and reverse primers (10 μM, 1 μL each), and 1μL of cDNA template. The cycling condition was as follows: 94 °C for 5min, then 30 cycles of 94 °C for 30 s, 55 °C for 30 s, and 72 °C for 30 s, and a final extension at 72 °C for 5 min. The PCR products were detected with 1.0% agarose gels. Then, the PCR products were purified using a Gel Extraction Kit (OMEGA Bio-Tek, USA) and were sequenced by Sangon Biotech (Shanghai, China). The sequences of PCR products were subjected to BLAST analysis. Furthermore, the samples were also examined for FMDV, SVDV, VSV, and VESV with the previously described RT-PCR methods [12,13], due to that all these pathogens could cause vesicular diseases. 

### 2.3. Virus Isolation

Virus isolation was performed in BHK-21 cells maintained in DMEM supplemented with 10% FBS (v/v). The vesicular fluid was diluted with sterile phosphate-buffered saline (PBS) and centrifuged at 12,000× *g* for 10 min. The supernatant was filtrated by 0.45 µm filters and then incubated with the BHK-21 cells. The inoculated cells were incubated at 37 °C in 5% CO_2_ and observed daily for cytopathic effect (CPE). When CPE was obvious in BHK-21 cells, the cells and medium were collected and the cultures were frozen and thawed repeatedly three times and centrifuged for 10 min at 10,000× *g* and 4 °C to collect the supernatant. Then, the presence of virus was examined by RT-PCR as above. For constructing the one-step growth curve, BHK-21 cells were inoculated with SVA strain at a multiplicity of infection (MOI) of 0.1, and the cell culture was collected every 4 hours for TCID_50_ determination. 

### 2.4. Sequencing and Phylogenetic Analyses

Eight pairs of primers were designed according to the SVA sequences in GenBank and used for amplification of the viral genome (Table 1). cDNA synthesis was carried out as above. PCR amplification was performed using Prime STAR^®^ HS (TAKARA, Dalian, China). The PCR products were purified and cloned into the pMD18-T vector. The inserted fragment in the pMD18-T vector was sequenced by Sangon Biotech (Shanghai, China). The overlapping sequences were assembled to generate the genomic sequence using the SeqMan program in the DNASTAR software (DNASTAR, Madison, WI, USA). The genome sequence of the isolate was subjected to BLAST analysis. Phylogenetic analysis was performed with the MEGA version 7.0 [14] using the neighbor-joining method and bootstrap validation with 1000 replications.

### 2.5. Pigs Challenge Experiment

The 100-day-old pigs were selected for virus challenge experiment. They were confirmed to be free of SVA, FMDV, SVDV, VSV, and VESV, detected with RT-PCR methods using the sera or oral swabs samples. Pigs were housed in Laboratory Animal Center of South China Agricultural University. Five pigs were randomly divided into two groups and maintained in separate rooms. The strict biosecurity protocols were followed to avoid cross contamination. Animals received food and water ad libitum during the experiment. Pigs were inoculated intranasally with 3 mL of SVA GD-ZYY02-2018 strain (10^8.25^ TCID_50_/mL) in the infected group (n = 3) and 3 mL of DMEM in the negative control group (n = 2). After the virus challenge, the clinical symptoms of animals were observed and recorded. The mental state and dietary status of each pig were observed. The rectal temperature of pigs was monitored. The viral load in blood was detected by real-time quantitative PCR as described previously [15]. Vesicular fluid and feces collected by cotton swab were tested for SVA by RT-PCR as above.

### 2.6. Immunization-Challenge Experiment

Inactivation of SVA strain with β-propiolactone (BPL) was performed as described previously [16] with slight modifications. Briefly, the SVA GD-ZYY02-2018 strain (10^8.25^ TCID_50_/mL) was inactivated with BPL at a final concentration of 0.05% (v/v). Then, the remaining BPL was hydrolyzed through incubating the BPL-treated virus suspension at 37 °C for 2 h. To confirm that the BPL-treated virus has been completely inactivated, the BPL-treated virus suspension was inoculated onto the BHK-21 cells. The inoculated cells were cultured followed by three consecutive passages of supernatants and were monitored daily for the presence of viral RNA and CPE. The BPL-treated virus was identified to be completely inactivated if no CPE was observed and no viral RNA was detected after three-time passages. To obtain the SVA experimental vaccine, the BPL-inactivated virus suspension was mixed and emulsified with adjuvant ISA 201 VG (SEPPIC, Paris, France) at a volume ratio of 1:1 and prepared for the immunization challenge experiment.

The 60-day-old pigs were selected for the immunization challenge experiment. They were confirmed to be free of SVA, FMDV, SVDV, VSV, and VESV. Pigs were housed in Laboratory Animal Center of South China Agricultural University. Six pigs were randomly divided into two groups and maintained in separate rooms. The strict biosecurity protocols were followed to avoid cross contamination. Animals received food and water ad libitum during the experiment. Three pigs in the vaccinated group were inoculated with 3 mL of inactivated SVA strain via intramuscular injection in the neck while three pigs in the non-vaccinated group were treated with 3 mL PBS in the same way. Three weeks later, the second immunization was performed in the same way as the first immunization. Blood samples were collected from pigs every week after the first immunization and the anti-SVA neutralizing antibody titers were determined by virus neutralizing antibody test (VNT) as reported previously [17] with slight modifications. Briefly, the pig serum was serially diluted and the serum of different dilutions was mixed with the equal volume of SVA GD-ZYY02-2018 solution (100 TCID_50_/100 μL). The mixture was incubated at 37 °C for 1 h and then added to the BHK-21 cells in the 96-well tissue culture plate. At 72 h post-infection (h.p.i.), the anti-SVA neutralizing antibody titers were determined and expressed as the reciprocal of the final serum dilution that could result in the neutralization of the SVA activity by 50%.

One week after the second immunization, all the vaccinated and non-vaccinated pigs were inoculated with 3 mL of SVA GD-ZYY02-2018 strain (10^8.25^ TCID_50_/mL) by intranasal routes. After the virus challenge, the clinical symptoms of animals were observed and recorded. The mental state and dietary status of each pig were observed. The rectal temperature of pigs was monitored after the virus challenge. The viral load in blood was detected by real-time quantitative PCR. Vesicular fluid and feces collected by cotton swab were tested for SVA by RT-PCR as above.

## 3. Results

### 3.1. A SVA Strain Was Isolated from Pig Herd with Vesicular Symptoms

**Figure 1:**
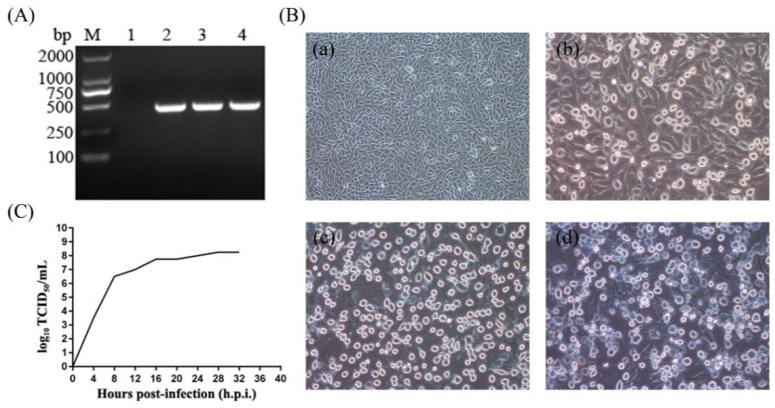
Isolation of SVA GD-ZYY02-2018 strain. (**A**) RT-PCR detection for SVA in vesicular lesion tissues. The size of the amplified products was consistent with the expected size (442 bp). M, DNA marker. Lane 1, negative control (H_2_O). Lanes 2~4, clinical samples. (**B**) Cytopathic effects (CPE) of the GD-ZYY02-2018 strain in infected BHK-21 cells. Changes in cell morphology, such as rounding, shrinkage, and nonadherence, were observed in infected BHK-21 cells. (**a**), 4 h.p.i.; (**b**), 8 h.p.i.; (**c**), 12 h.p.i.; (**d**), 16 h.p.i. (**C**) One-step growth curve of the GD-ZYY02-2018 strain.

Clinical samples from a pig farm with vesicular symptoms in Guangdong province were collected. RT-PCR was used to detect suspected agents (SVDV, VSV, VESV, FMDV, and SVA) that could cause vesicular diseases. The size of the PCR products amplified by primers SVA-F and SVA-R was consistent with the expected size (442 bp) (Figure 1A). The sequence of the PCR products shared high homology with recent Chinese SVA strains through the BLAST analysis (data not shown), which meant that the clinical samples were SVA-positive. In addition, the clinical samples were negative for other vesicle-associated pathogens including SVDV, VSV, VESV, and FMDV (data not shown).

Subsequently, an SVA strain was successfully isolated from SVA-positive clinical samples. CPE characterized by rounding, shrinkage, and nonadherence in BHK-21 cells was obvious at 8 h.p.i. by incubating the third passage of virus solutions (Figure 1B). Furthermore, a one-step growth curve was constructed to describe the proliferation dynamics of the isolated strain (Figure 1C).

### 3.2. Characterization of the SVA Genome Sequence

The isolated SVA strain was designated as GD-ZYY02-2018. The genome sequence of the GD-ZYY02-2018 strain was determined and deposited in the GenBank under accession number (MT840202). The result of BLAST analysis on NCBI showed that GD-ZYY02-2018 strain shared a high homology (99.07~99.19%) with Chinese SVA strains such as CHhb17 (China, 2017), SVA-CHN-01-2017 (China, 2017), SVA-CHN-02-2017 (China, 2017), GD01-2017 (China, 2017), and GD03-2017 (China, 2017).

**Figure 2:**
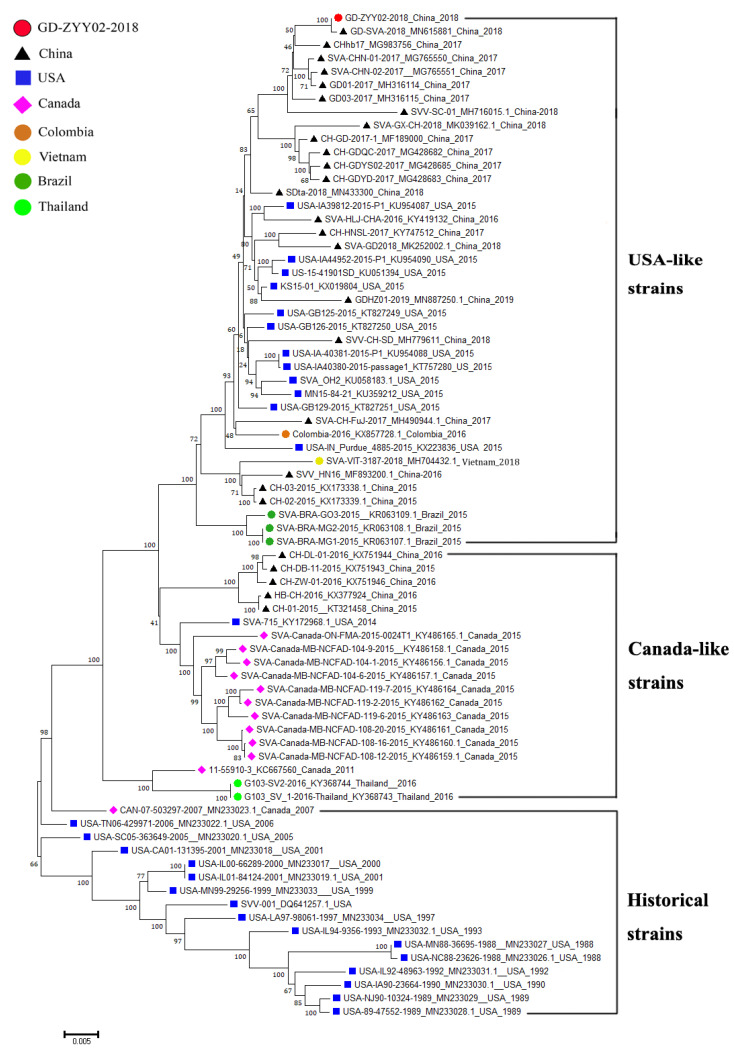
Phylogenetic analysis based on the genome sequences of GD-ZYY02-2018 strain and reference SVA strains. Both historical strains (1988–2010) and contemporary strains (2011–2019) were selected as reference SVA strains. The phylogenetic tree was constructed with MEGA version 7.0 using the neighbor-joining method, with bootstrap validation using 1000 replications. The GD-ZYY02-2018 strain was marked with a red circle. The results of phylogenetic analysis show that the GD-ZYY02-2018 strain belongs to the USA-like strains and has a close genetic relationship with recent Chinese SVA strains.

A phylogenetic tree based on whole-genome sequences of historical (detected between 1988–2010) and contemporary SVA strains (detected between 2011–2019) (Table S1) was constructed (Figure 2). Historical SVA strains from the USA and Canada were clustered together. Contemporary SVA strains had a distant relationship with historical SVA strains and could be classified into two types, including the USA-like strains and Canada-like strains. According to the phylogenetic analysis, SVA strains from China could be divided into two categories including the USA-like strains and Canada-like strains and Chinese SVA strains isolated after 2017 were found to belong to the USA-like strains. The GD-ZYY02-2018 strain also belonged to the USA-like strains because the GD-ZYY02-2018 strain was clustered together with the USA-like strains. As showed in the phylogenetic tree (Figure 2), GD-ZYY02-2018 strain had a close genetic relationship with Chinese SVA strains such as GD-SVA-2018 (China, 2018), CHhb17 (China, 2017), SVA-CHN-01-2017 (China, 2017), SVA-CHN-02-2017 (China, 2017), GD01-2017 (China, 2017), and GD03-2017 (China, 2017).

### 3.3. Pathogenicity of SVA GD-ZYY02-2018 Strain in Pigs

**Figure 3:**
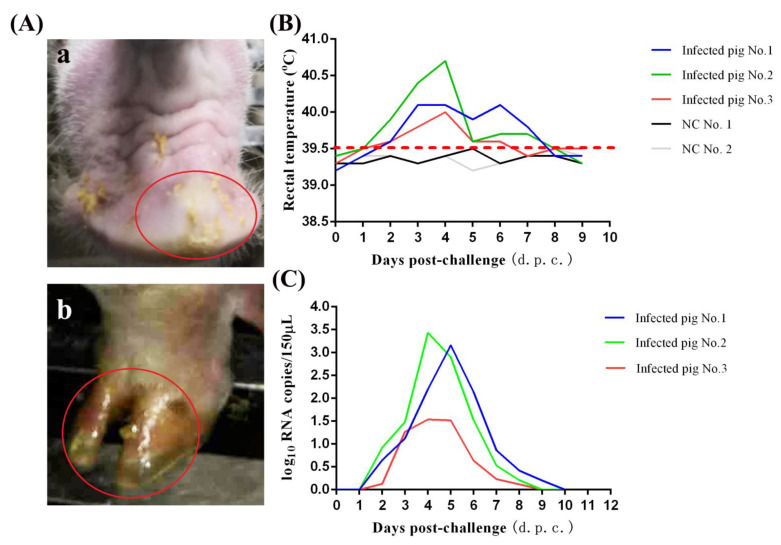
Pathogenicity of GD-ZYY02-2018 strain on pigs. (**A**) Clinical outcome post-challenge of GD-ZYY02-2018 strain. (**a**), Vesicular lesions at the snout were observed in the infected pigs. (**b**), Ulcerative lesions at the hooves were observed in the infected pigs. (**B**) Body temperature changes in pigs after GD-ZYY02-2018 infection. (**C**) Viremia levels as determined by RT-qPCR in serum samples collected at the indicated times post-challenge of GD-ZYY02-2018 strain.

Challenge experiment was performed in 100-day-old pigs. All the pigs survived after viral infection. Vesicular lesions could be observed on the snout of infected pigs at 2~3 days post-challenge (d.p.c.) (Figure 3A). Vesicular lesions maintained about 5 days, which evolved through different stages including erythema, fluid-filled vesicles, ruptured vesicles, skin ulcers, scabby lesions, and finally normal skin. A slight increase in body temperature could be detected after challenge (Figure 3B). The viremia could be detected in all infected pigs. Viral loads in the serum peaked at 3~4 d.p.c. Then, the viremia levels declined rapidly and disappeared at 9~10 d.p.c. (Figure 3C). Viral nucleic acid was present in the vesicular lesions and also present in feces (data not shown). SVA could be successfully re-isolated from vesicular lesions and feces of challenged pigs using BHK-21 cells (data not shown).

### 3.4. Protective Efficacy of Inactivated GD-ZYY02-2018 Strain

**Figure 4:**
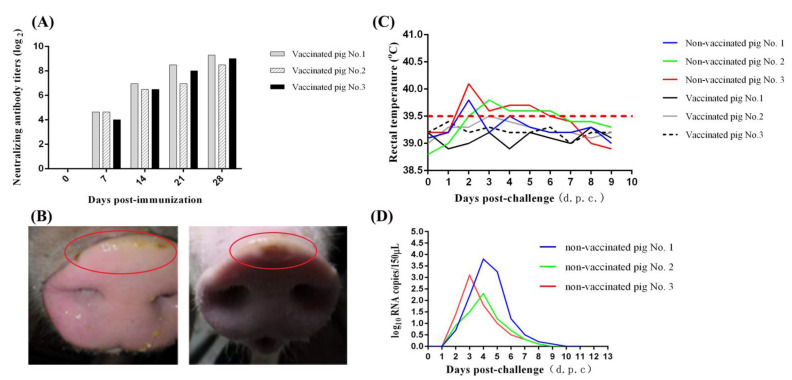
Protection efficiency of inactivated GD-ZYY02-2018 strain. (**A**) Anti-SVA neutralizing antibody titers of serum in pigs post-immunization. (**B**) Clinical outcome post-infection of GD-ZYY02-2018 strain in non-vaccinated pigs. Vesicular lesions were observed on the snout of non-vaccinated pigs, but not in vaccinated pigs (data not shown). (**C**) Body temperature changes in pigs after infection with GD-ZYY02-2018 strain in the immunization challenge experiment. (**D**) Viremia levels as determined by RT-qPCR in serum samples collected at the indicated times post-challenge of GD-ZYY02-2018 strain in the immunization challenge experiment.

To determine the protective efficacy of inactivated SVA GD-ZYY02-2018 strain, the immunization challenge experiment was carried out. Pigs vaccinated with inactivated GD-ZYY02-2018 strain could produce a high level of anti-SVA neutralizing antibody (Figure 4A) and no abnormal reaction was observed in vaccinated pigs. After two immunization, all pigs were challenged with the GD-ZYY02-2018 strain. Pigs both in vaccinated and non-vaccinated group survived after viral infection. Vesicular lesions could be observed on the snout of pigs in the non-vaccinated group, but not in the vaccinated group (Figure 4B). A slight increase in body temperature was observed in non-vaccinated pigs, but not in vaccinated pigs (Figure 3C). The viremia could be detected in non-vaccinated pigs, but not in vaccinated pigs (Figure 4D). Viral nucleic acid was present in feces from non-vaccinated pigs, but not from vaccinated pigs (data not shown). The result showed that the inactivated GD-ZYY02-2018 strain could protect finishing pigs from the challenge of the GD-ZYY02-2018 strain. 

## 4. Discussion

In the present study, phylogenetic analysis based on genome sequences of SVA strains from different regions was carried out. The results of the phylogenetic analysis suggested that the USA-like SVA strains were predominant in China, which was consistent with the previous studies [18,19]. Besides in China, the USA-like strains were also found in the USA, Brazil, Vietnam, and Colombia. Due to the wide spread of the USA-like strains, we speculated that there was a high risk that the USA-like strains might spread to SVA-free areas through trade and international transport. Thus, it was necessary to further study how to curb the spread of the USA-like strains. GD-ZYY02-2018, an USA-like strain, was isolated in this work. Studies on the GD-ZYY02-2018 strain will help to understand the biological characteristics of the USA-like strains and prevent and control the infection of the USA-like strain.

Vaccination plays an important role in the prevention and control of animal contagious diseases. SVA infection has affected the productivity of the pig herd and caused economic losses to the pig industry. The long-termed circulation, spread, and variation of SVA will make it more difficult and complicated to control the disease. Thus, we think that the development of SVA vaccine is meaningful and can curb the prevalence of SVA. However, there were few reports about the research of SVA vaccine [17,20,21] and no commercial vaccine for SVA was available. Thus, it was necessary to continue the research of SVA vaccine. First of all, it was important to establish an animal model of SVA infection in finishing pigs, which would be conducive to reveal the pathogenic mechanism of SVA and provide a suitable animal infection model for vaccine research. With reference to previous studies about the pathogenicity of SVA [19,22,23], we established the SVA infection model for finishing pigs. Results of challenge experiment showed that the GD-ZYY02-2018 strain could successfully infect finishing pigs. The infected pigs displayed a mild fever and developed characteristic vesicular symptoms. The viremia could be detected in infected pigs at 2 d.p.c. and disappeared at 9~10 d.p.c., demonstrating that viruses in pigs could be cleared by the immune system. The virus could be re-isolated from vesicular lesions and feces, indicating that infected pigs could release the virus into the environment through vesicular lesions and feces. In short, the vesicular disease caused by SVA was successfully reproduced in finishing pigs in this work.

After establishing the SVA infection model, we further carried out the research of SVA vaccine. Considering that inactivated vaccines do not contain live pathogens and are safe for less risk of inducing the disease, we preliminarily studied the protective efficacy of inactivated SVA strain in this work. The GD-ZYY02-2018 was proliferated in BHK-21 cells and inactivated by BPL. Preliminary results of the immunization challenge experiment showed that experimental vaccine based on the inactivated SVA had good safety in finishing pigs and significantly protected finishing pigs against virus challenge, which suggested that GD-ZYY02-2018 strain was a potential vaccine candidate. However, further studies are needed to verify the safety and protection efficiency of the inactivated SVA vaccine. The most important work is to verify whether the inactivated SVA vaccine could protect pigs against the challenge of heterologous strains. Then, more details are needed to be considered. For example, the number of experimental animals should be increased. The optimized dose of the vaccine should be investigated.

## 5. Conclusions

In the present study, an USA-like strain (GD-ZYY02-2018) was isolated from a pig herd with vesicular symptoms. It had a close genetic relationship with recent Chinese SVA strains. Vesicular symptoms caused by the GD-ZYY02-2018 strain were successfully reproduced in finishing pigs. Our study also preliminarily confirmed that the inactivated GD-ZYY02-2018 strain could protect finishing pigs against virus challenge. This work would be useful for studying the pathogenesis of SVA infection and controlling this disease in the future.

## Figures and Tables

**Table 1:** Primers for the amplification of Senecavirus A (SVA) genome sequence.

Primers	Sequences (5′-3′)
SVA-0-F	TTTGAAATGGGGGGCTGG
SVA-0-R	CGAGCCGTGGATATTCAA
SVA-1-F	GCACAGAGGAGCAACATC
SVA-1-R	GATGTCCAGTCCAAGTTGT
SVA-2-F	TAACCGACCTCTTACAACTG
SVA-2-R	TGAGACCACCGTGACTTC
SVA-3-F	CAGCAGGACGATGGTTAC
SVA-3-R	GGAGGCGGTTCTACAGTA
SVA-4-F	ACCTGGAGGAAGTATGTGA
SVA-4-R	GTCTCTTCTCGGTCTGTATC
SVA-5-F	GGCGTTGGGTAGAGTTC
SVA-5-R	GAGTAGTCACCGTCTAAGAAT
SVA-6-F	GCAAGGACTGATGACTGA
SVA-6-R	CACCGTAGGCGATGATAT
SVA-7-F	CGCCAAGTTTCAATCCCATC
SVA-7-R	TCCAGGTCAGTCGAACAA

## Data Availability

Data are contained within the article and supplementary material (Table S1: Reference SVA strains selected for constructing the phylogenetic tree).

## Supplementary Materials

The following are available online at https://www.mdpi.com/2075-1729/11/2/157/s1, Table S1: Reference SVA strains selected for constructing the phylogenetic tree.
