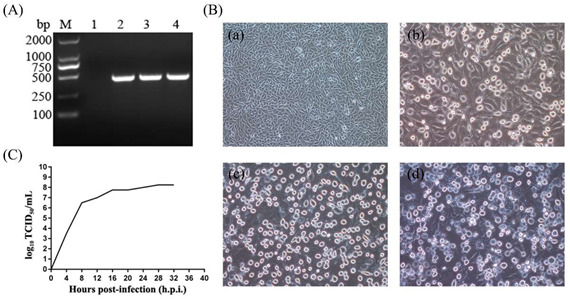# Correction: Li et al. Preliminary Evaluation of Protective Efficacy of Inactivated Senecavirus A on Pigs. *Life* 2021, *11*, 157

**DOI:** 10.3390/life14121545

**Published:** 2024-11-25

**Authors:** Yuwan Li, Yangyi Zhang, Yingxin Liao, Yawei Sun, Yang Ruan, Chenchen Liu, Mengru Zhang, Fangfang Li, Xiaowen Li, Shuangqi Fan, Lin Yi, Hongxing Ding, Mingqiu Zhao, Jindai Fan, Jinding Chen

**Affiliations:** 1 College of Veterinary Medicine, South China Agricultural University, Guangzhou 510642, China; waner20191028012@stu.scau.edu.cn (Y.L.); zhangyy6@mail.sustech.edu.cn (Y.Z.); yxliao@soil.gd.cn (Y.L.); syw18530494979@stu.scau.edu.cn (Y.S.); ruanyang@stu.scau.edu.cn (Y.R.); liuchenchen@stu.scau.edu.cn (C.L.); zmr15625156296@stu.scau.edu.cn (M.Z.); fangfangli@stu.scau.edu.cn (F.L.); xiaowenlee@stu.scau.edu.cn (X.L.); shqfan@scau.edu.cn (S.F.); yilin@scau.edu.cn (L.Y.); dinghx@scau.edu.cn (H.D.); zmingqiu@scau.edu.cn (M.Z.); 2 Guangdong Laboratory for Lingnan Modern Agriculture, College of Veterinary Medicine, South China Agricultural University, Guangzhou 510642, China; 3 Key Laboratory of Zoonosis Prevention and Control of Guangdong Province, Guangzhou 510642, China

## Error in Figure

In the original publication, there was a mistake in Figure 1B(b,c) as published. Upon thorough examination of the original data, we found that this was due to an error in selecting the correct images for assembly using Photoshop software, and there was a lack of careful checking in the later stages. The corrected, revised Figure 1 appears below. The authors state that the scientific conclusions are unaffected. This correction was approved by the Academic Editor. The original publication has also been updated.