# Supplementary material for: Preliminary Evaluation of Protective Efficacy of Inactivated Senecavirus A on Pigs
Source: Life (Basel). 2021 Feb 18;11(2):157. doi: 10.3390/life11020157 (PMC7922346; doi:10.3390/life11020157)
Supplement: Supplementary file 1 [file life-11-00157-s001.pdf]

Article

Preliminary Evaluation of Protective Efficacy of Inactivated Senecavirus A on Pigs

Yuwan Li [1,2,3,†], Yangyi Zhang [1,2,3,†], Yingxin Liao [1,2,3,†], Yawei Sun [1,2,3], Yang Ruan [1,2,3], Chenchen Liu [1,2,3], Mengru Zhang [1,2,3], Fangfang Li [1,2,3], Xiaowen Li [1,2,3], Shuangqi Fan [1,2,3], Lin Yi [1,2,3], Hongxing Ding [1,2,3], Mingqiu Zhao [1,2,3], Jindai Fan [1,2,3,*] and Jinding Chen [1,2,3,*]

1 College of Veterinary Medicine, South China Agricultural University, Guangzhou 510642, China; waner20191028012@stu.scau.edu.cn (Yu.L.); zhangyy6@mail.sustech.edu.cn (Y.Z.); yxliao@soil.gd.cn (Yi.L.); syw18530494979@stu.scau.edu.cn (Y.S.); ruanyang@stu.scau.edu.cn (Y.R.); liuchenchen@stu.scau.edu.cn (C.L.); zmr15625156296@stu.scau.edu.cn (Me.Z.); fangfangli@stu.scau.edu.cn (F.L.); xiaowenlee@stu.scau.edu.cn (X.L.); shqfan@scau.edu.cn (S.F.); yilin@scau.edu.cn (L.Y.); dinghx@scau.edu.cn (H.D.); zmingqiu@scau.edu.cn (Mi.Z.)

2 Guangdong Laboratory for Lingnan Modern Agriculture, College of Veterinary Medicine, South China Agricultural University, Guangzhou 510642, China

3 Key Laboratory of Zoonosis Prevention and Control of Guangdong Province, Guangzhou 510642, China

† These authors contributed equally to this work

* Correspondence: fanjindai@stu.scau.edu.cn (J.F.); jdchen@scau.edu.cn (J.C.); Tel.: +86-20-8528-8017 (J.F.); Tel.: +86-20-8528-8017 (J.C.)

Supplementary Materials:

Table S1. Reference SVA strains selected for constructing the phylogenetic tree.

Isolate name	GenBank No.	Country of isolation	Year of isolation
USA/NC88-23626/1988	MN233026	USA	1988
USA/MN88-36695/1988	MN233027	USA	1988
USA/89-47552/1989	MN233028	USA	1989
USA/NJ90-10324/1989	MN233029	USA	1989
USA/IA90-23664/1990	MN233030	USA	1990
USA/IL92-48963/1992	MN233031	USA	1992
USA/IL94-9356/1993	MN233032	USA	1993
USA/LA97-98061/1997	MN233034	USA	1997
USA/MN99-29256/1999	MN233033	USA	1999
USA/IL00-66289/2000	MN233017	USA	2000
USA/CA01-131395/2001	MN233018	USA	2001
USA/IL 01-84124/2001	MN233019	USA	2001
SVV-001	DQ641257	USA	2002
USA/SC05-363649/2005	MN233020	USA	2005
USA/TN06-429971/2006	MN233022	USA	2006
CAN/07-503297/2007	MN233023	Canada	2007
11-055910/2011	KC667560	Canada	2011
SVA-715	KY172968	USA	2014
CH-01-2015	KT321458	China	2015
CH-02-2015	KX173339	China	2015
CH-03-2015	KX173338	China	2015
CH-DB-11-2015	KX751943	China	2015
KS15-01	KX019804	USA	2015
MN15-84-21	KU359212	USA	2015
SVA-OH2	KU058183	USA	2015
US-15-41901SD	KU051394	USA	2015

USA-GB125-2015	KT827249	USA	2015
USA-GB126-2015	KT827250	USA	2015
USA-GB129-2015	KT827251	USA	2015
USA-IA39812-2015-p1	KU954087	USA	2015
USA-IA40380-2015-passage1	KT757280	USA	2015
USA-IA40381-2015-P1	KU954088	USA	2015
USA-IA44952-2015-P1	KU954090	USA	2015
USA-IN Purdue 4885-2015	KX223836	USA	2015
SVA-BRA-GO3-2015	KR063109	Brazil	2015
SVA-BRA-MG1-2015	KR063107	Brazil	2015
SVA-BRA-MG2-2015	KR063108	Brazil	2015
SVA-Canada-MB-NCFAD-104-1-2015	KY486156	Canada	2015
SVA-Canada-MB-NCFAD-104-6-2015	KY486157	Canada	2015
SVA-Canada-MB-NCFAD-104-9-2015	KY486158	Canada	2015
SVA-Canada-MB-NCFAD-108-12-2015	KY486159	Canada	2015
SVA-Canada-MB-NCFAD-108-16-2015	KY486160	Canada	2015
SVA-Canada-MB-NCFAD-108-20-2015	KY486161	Canada	2015
SVA-Canada-MB-NCFAD-119-2-2015	KY486162	Canada	2015
SVA-Canada-MB-NCFAD-119-6-2015	KY486163	Canada	2015
SVA-Canada-MB-NCFAD-119-7-2015	KY486164	Canada	2015
SVA-Canada-ON-FMA-2015-0024T1	KY486165	Canada	2015
CH-DL-01-2016	KX751944	China	2016
CH-ZW-01-2016	KX751946	China	2016
HB-CH-2016	KX377924	China	2016
SVA-HLJ-CHA-2016	KY419132	China	2016
SVV-HN16	MF893200	China	2016
G103-SV-1-2016	KY368743	Thailand	2016
G103-SV2-2016	KY368744	Thailand	2016
Colombia-2016	KX857728	Colombia	2016
CH-GD-2017-1	MF189000	China	2017
CH-GDQC-2017	MG428682	China	2017
CH-GDYD-2017	MG428683	China	2017
CH-GDYS02-2017	MG428685	China	2017
CHhb17	MG983756	China	2017
CH-HNSL-2017	KY747512	China	2017
GD01-2017	MH316114	China	2017
GD03-2017	MH316115	China	2017
SVA-CH-FuJ-2017	MH490944	China	2017
SVA-CHN-01-2017	MG765550	China	2017
SVA-CHN-02-2017	MG765551	China	2017
GD-SVA-2018	MN615881	China	2018
SDta-2018	MN433300	China	2018
SVA-GX-CH-2018	MK039162	China	2018
SVV-CH-SD	MH779611	China	2018
SVA-GD-2018	MK252002	China	2018
SVA-VIT-3187-2018	MH704432	Vietnam	2018
SVV-SC-01	MH716015	China	2018
GDHZ01-2019	MN887250	China	2019
